# Belladonna in Hernia

**Published:** 1858-01

**Authors:** 


*Belladonna in Hernia.*—In the Gazette Hebdomadaire of the 9th of October, we notice the report of a case of inguinal hernia which was relieved after taxis had failed, by the administration of the Ext. Belladonna, in three or four grain doses every half hour. The Tinct. of Belladonna was also employed locally by means of a flaxseed poultice.

In the same Journal we find the report of another case in which Belladonna was administered with very great relief to the patient. In this there was no protrusion of the bowel, but from the symptoms, obstinate constipation, great pain, stercoraceous vomiting, &c., it was evident that there was complete occlusion of the bowel. The Belladonna in this case was employed after an operation for artificial anus had been decided upon. The remedy was used in the form of ointment by friction, and as soon as complete intoxication was induced, its good effect was perceptible.

